# Realist evaluation of the AKU-SONAM mentorship program

**DOI:** 10.1371/journal.pone.0316816

**Published:** 2025-01-24

**Authors:** Rehana Rehman, Quratulain Javaid, Saira Khalid, Tazeen Saeed Ali, Rahila Ali

**Affiliations:** 1 Department of Biological & Biomedical Sciences, Aga Khan University, Karachi, Pakistan; 2 Bahria University Health Sciences, Campus, Karachi, Pakistan; 3 College of Nursing Armed Forces Postgraduate, Medical Institute (AFPGMI), Rawalpindi, Pakistan; 4 School of Nursing and Midwifery, Aga Khan University, Karachi, Pakistan; 5 Department for Educational Development, Aga Khan University, Karachi, Pakistan; Bilawal Medical College, Liaquat University of Medical and Health Sciences, PAKISTAN

## Abstract

**Background & objectives:**

The context, mechanism, and outcome (CMO) framework is meant to identify specific contextual factors (C) related to organizational and program structure that trigger certain mechanisms (M) involving the unique characteristics of a program, leading to specific outcomes (O). The purpose of this study was to explore the contextual underpinnings, operational processes, and resultant effects of the faculty mentorship program at AKU-SONAM. This exploration involved the context in terms of organizational culture, mechanisms examining processes such as communication between mentors and mentees, quality of relationships, the challenges encountered, and the program’s adaptability to cope up while, outcomes encompassed improvements in interpersonal relationships, career advancement, and skill development.

**Methods:**

A qualitative exploratory study was conducted at AKU-SONAM, involving in-depth interviews (IDIs) with program leadership and administrators, as well as focus group discussions (FGDs) with mentors and mentees. A semi-structured interview guide, comprising open-ended, introductory, probing, and concluding questions, was developed, validated, and reviewed by experts to ensure its effectiveness and relevance. The process included transcription of interviews, verification through member checks, and thematic analysis where codes and categories were identified and themes, were developed guided by the CMO (Context-Mechanism-Outcome) configuration framework.

**Results:**

Three IDIs were conducted with administrators and leadership while two FGD were conducted independently with mentors and mentees. Themes for context, mechanism, and outcome were identified. The theme “*Navigating Mentorship Challenges in the Professional Landscape*” described the Context with subthemes: “Existence of mentorship culture”, Origins and Evolution of Mentoring” “Mentor’s pool, Availability of resources and Mentorship to Seniors. ‘*Synergies in Professional Development*: *From Interdepartmental Collaboration to Mentor-Mentee Connections’* was the theme for mechanisms with subthemes; ‘Fostering Interdepartmental Collaboration’ ‘Building Connections’, ‘sharing of thoughts’, Work Driven Model, Mentor mentee pairing and Selection of mentor. Theme: ‘*Cultivating Excellence in the Orchard for Outcome*’ emerged from ‘Exchange of Insights in the Orchard of Mentorship’ and ‘Skills Development” constituted the Outcome theme.

**Conclusions:**

The mentorship program at AKU-SONAM facilitated mentors and mentees to establish connections and exchange ideas. While the mentor-mentee pairing process was clearly outlined, participants emphasized the need to address the challenges encountered by mentees. Although the outcomes were not explicitly clarified, the program contributed to the advancement of the professional development of mentees at AKU-SONAM.

## Introduction

Mentoring is a distinctive developmental relationship that supports the comprehensive growth of both the mentor and mentee, encompassing skill acquisition, knowledge expansion, and emotional resilience [1]. Well-structured mentorship programs expedite knowledge dissemination, optimize recruitment and retention strategies, nurture a culture of ongoing learning and development, and enhance the quality of teaching, service, and research [2, 3]. Effective mentorship programs thus ensuring the individual growth and development of mentors and mentees through the involvement of all stakeholders particularly the institutions [2, 4]. This consequently advances the quality of healthcare services and research within the organization.

The Faculty Mentorship Program at the Aga Khan University School of Nursing and Midwifery (AKU-SONAM) was established in 2002 with the aim to provide comprehensive support and guidance to newly appointed nurse faculty members [5]. The program focuses on various aspects of professional development, including teaching, practice, research, and administration. A wide variety of mentorship models exist in different universities [6] which provide a structured framework for mentoring relationships, guiding the interactions and goals between mentors and mentees to ensure effectiveness of the program [7]. Among the various models, one-on-one Mentoring, the traditional mentoring model was employed by this program which tends to build the ease of discussion between the mentor and mentees [8].

Though formal mentoring programs directly benefit academic health care institutions, it is important to confirm that the programs use recommended components and that the desired outcomes are evaluated periodically [7]. **Realistic Evaluation** (RE) Is a theory-driven model of evaluation and provides a realistic and adaptable framework for evaluating and designing effective programs. It is guided by the concept that participants engaged in an intervention operate within a distinct social context, which shapes a participant’s behavior consequently influencing the outcome of the intervention [9].

AKU-SONAM demonstrates a commitment to a rigorous and comprehensive evaluation of the faculty mentorship program. Therefore, a realist lens to mentorship evaluation was applied by exploring the context (C) of this program which comprised of organizational culture, circumstances, and factors that influenced the behavior and attitude of participants towards the mentorship program. The (M) Mechanism included all the processes such as communication between mentors and mentees, quality of relationships, the challenges encountered, and the program’s adaptability to cope with the issues. The outcome (O) in the CMO framework is explicit and implicit of results in the observed phenomenon. This approach will thus help to identify areas for improvement and ensure that the program is meeting the needs of their faculty members and provide recommendations to academic leadership for improvements in indented outcomes.

## Methods

- Ethics approval and consent to participate:

All methods were carried out following relevant guidelines and regulations. Participation in the study was voluntary. Informed Consent was taken before the in-depth interviews and the focus group discussions.

A qualitative exploratory study was conducted in 2021–2023 after ethical approval from the institution (ERC—2021-6127-17832).

The participants included in the study were from all levels of the hierarchy comprising leaders, administrators, mentors, and mentees. The interview guides were prepared after a relevant literature search. The interview guide for IDI was focused on the specific role of leadership and administrators in the program (Appendix I) whereas pertinent questions regarding roles responsibilities and challenges faced by mentors and mentees were included in the interview guide FGD (Appendix II). Before execution, both guides were reviewed by the research team and representatives from the Department for Educational Development. Seven participants from AKU-SONAM took part in the study, comprising two individuals from the administration, one from leadership, two mentors, and two mentees. Three in-depth interviews were conducted with the administrators and leadership from 03/11/2021 to 08/11/2021, while two FGDs were held with the mentors and mentees on 24/12/2021 and 30/12/2021. Permission was taken from all the participants before the initiation of the discussion. Informed consent was signed, and one copy of consent was handed over to the individual participants. Field notes were taken by the primary investigator. All interviews were conducted by a research assistant appointed for this job. A reflective log was maintained to avoid researcher bias. Each interview/FGD lasted for almost 40–60 minutes. They were audiotaped after participants’ approval. The collection process stopped with the saturation of data as there was repetition of responses and no more meaningful information was obtained. Thematic analysis of the results was conducted to understand patterns and themes were developed to classify the data [10].

All verbatim were transcribed. Codes, categories, and an inductive approach to thematic analysis was done through a rigorous process of member check, cross-verification, consulting field notes, and reflective logs.

## Results

**Fig 1:**
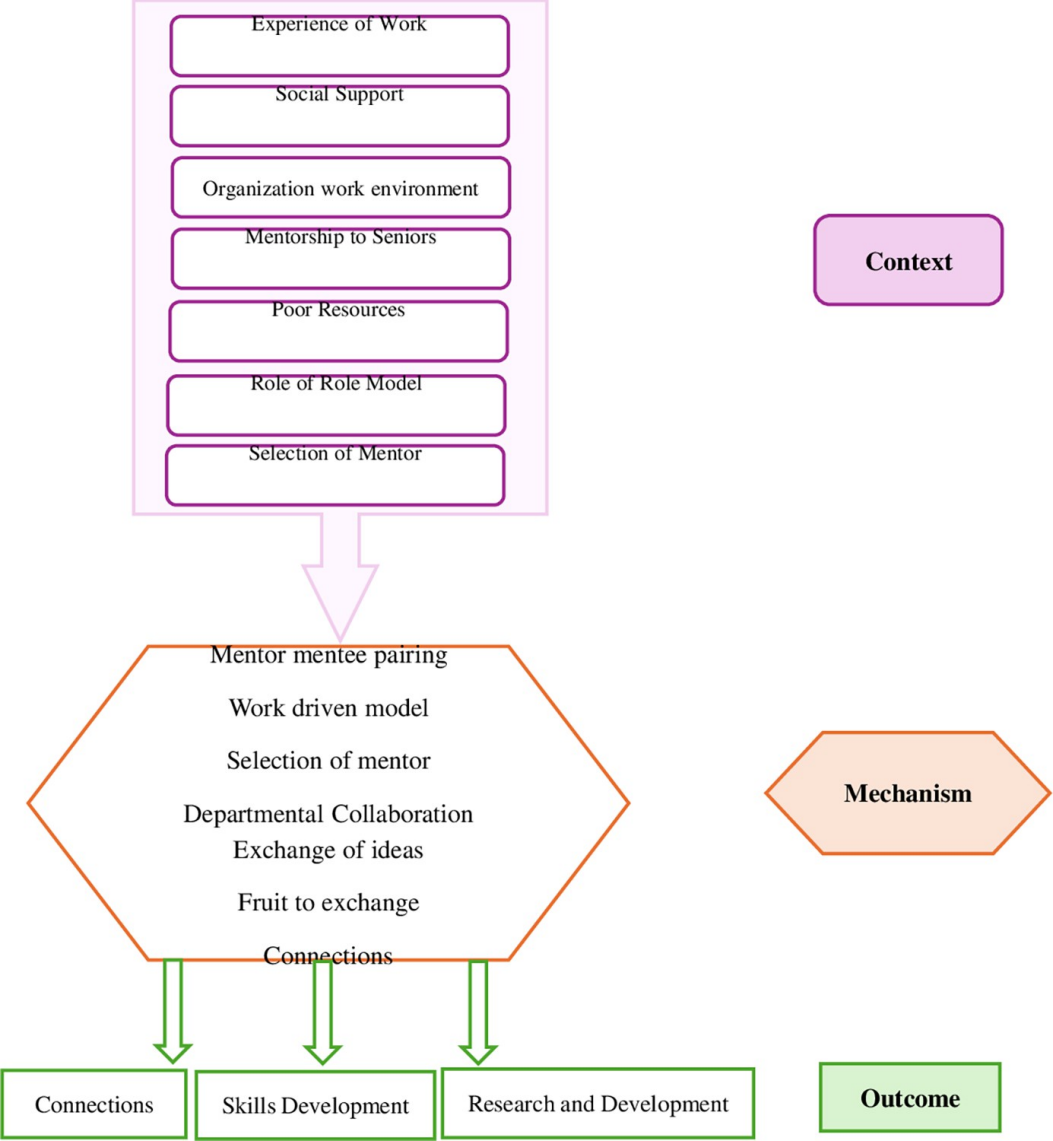
Context-mechanism-outcome configuration/ conceptual framework on realist evaluation AKU-SONAM.

Three major themes emerged which were later categorized and explained under the notions of Context, Mechanism, and Outcome (Fig 1).

Table 1 displays the verbatims, subthemes, and overarching themes. Three themes for context, mechanism, and outcome were identified. Context was covered by the theme: “Mentorship Program Evaluation: A Comprehensive Assessment of Effectiveness*”* which was based on; *mentorship* culture, the evolution of mentoring, the Mentor Pool, mentorship to seniors, the feedback system, and availability of resources. The recognized theme for ‘mechanisms’ in the AKU-SONAM mentorship program was “*Synergies in Professional Development*: *From Interdepartmental Collaboration to Mentor-Mentee Connections”* which comprised of mentor-mentee pairing, mentor-mentee dynamics, work-driven model, interdepartmental collaboration and sharing of ideas. The exchange of insights in mentoring, skills development, and research development constituted the ‘outcome’ theme; “*Cultivating Excellence in the orchard of mentorship*”

**Table 1:** 

**Domain 1: Context**	
Mentorship Program Evaluation: A Comprehensive Assessment of Effectiveness	
Subthemes	Illustrative quotes
Existence of mentorship culture	• *When I joined*, *I was not assigned formal mentorship*, *however*, *I was given full support from the senior faculty*. • *Social support from peers in the mentorship program is worth appreciating*
Origins and Evolution of Mentoring	• *The seed of mentoring at the School of Nursing was sown much earlier than 2015*, *and significant work has been done in the past two years*. • *We are motivated enough to plan a mentorship unit at AKU-SONAM soon*. • *Another initiative is the introduction of the mentorship award*, *which marks a positive progression of our efforts*. • *The performance in clinical and academics is currently being assessed in a semi-structured way*. • *To have structured feedback*, *the guidelines*, *flow or process should be available in writing*
Mentor’s pool	• *The mentorship committee at SONAM has developed a pool of faculty members who agreed to serve as mentors*. • *The list is available with the administrators for the mentees to select mentors of their specialty like research*, *teaching*, *or clinical*. • *Once on board the novice is given a probationary period of four to six weeks to establish a rapport with the mentor*, *learn all the details*, *and get familiarized with the policies* • *Mentees should have a balance of responsibility and freedom to portray their mentors*.
Mentorship to Seniors	• *We need to explore the development of effective mentorship programs tailored for senior faculty*
Feedback system	• *The mentoring feedback system needs revision* • *structured guidelines should be provided*
Availability of resources	• *Fiscal resources are equally important as human resources*. • *We need financial resources to run the program efficiently and effectively*
**Domain 2: Mechanism**	
Synergies in Professional Development: From Interdepartmental Collaboration to Mentor-Mentee Connections	
Mentor mentee pairing	• *We maintain a list of mentors who must have over two years of experience and hold the rank of associate professor or higher*. • *During the pairing process*, *we match individuals based on their specialties and continue to monitor their progress after 4–6 weeks*. • *Throughout the probation period*, *mentees provide regular informal feedback*, *which is validated by their mentors*. *Following this period*, *both mentors and mentees are encouraged to reflect on their experiences*. • *This gives them the right direction to work and relate to the particular entity* • *Quote from mentees* • *The mentorship program has helped in my professional growth though I think that mentees should be given a choice for selection of mentors*
Mentor mentee dynamics	• *I have been mentoring for years and feel that mentees should have the responsibility for their learning and growth*. • *Although the whole process works in collaboration*, *still it is the mentee who requires support and guidance for progress and career development*. • *I would appreciate it if mentees came forward and take the initiative to seek guidance and support*. • *Mentorship is a two-way learning process*, *and I appreciate the role of the mentorship committee to facilitate this collaboration*
Work driven model	• *Keeping in mind the protected time required for scheduling mentor-mentee meetings*, *we propose a work-driven model of mentorship*. • *In this approach*, *the concept of formal meetings or feedback is replaced by shared workspace*, *fostering collaboration*, *coordination*, *and ongoing feedback*. • *I think this is a more sustainable solution*
Fostering Interdepartmental Collaboration and Building Connections	• *Mentorship should not be limited to a particular entity or a task …* • *There should be a collaboration of AKU-SONAM with AKU-MC*, *the Department of Educational Development (DED)*, *and the Institute of Educational Development (IED) at AKU*. • *We can formulate a mentorship committee of approximately 20 persons*, *five from each entity with a chair and co-chair*. • *They should meet quarterly or monthly through get together or webinars and brief one another on recent advancements done to uplift the program*
Sharing of ideas	• *We have good resources of internal mentors*, *however*, *the need for external mentors for an external worldview is desired*. • *The current status is the research mentorship in the Queen Elizabeth scholarship program …* • *For the past 15 years we have had research mentorship with external collaborators*. *I have learned from my mentors that every faculty*, *at every level*, *should have a mentor which can be an internal or an external mentor*. *I have mentorship from academicians*, *educationists*, *and researchers separately*. *This is how we can get mentorship components as per our needs from different avenues*
**Domain 3: Outcome**	
Theme: Cultivating Excellence in the orchard of mentorship	
Exchange of Insights in the Orchard of Mentoring	• *If we can have an exchange of mentors*, *between SONAM*, *medical college*, *DED*, *and IED which is likely to happen in the future we can select a mentor from another entity as per our needs* • *At this time*, *I am sharing a bigger picture that can fulfill our goal and make a big dream come true*, *not clear*
Skills development	• *In the absence of defined outcome measures*, *I found it challenging to assess the improvements in the mentee after a probation period of 4–6 weeks*. • *We don’t have a formal tool to assess the process of learning or to gauge the learning of mentees on an individual basis*
Research and Development	• *We have research themes like maternal and newborn*, *adolescent health*, *mental health*, *and geriatric palliative care so that people align themselves for the research within the respective theme*. • *The research office provides mentorship and guidance to all the faculty members through the senior leadership forum”*

## Discussion

At AKU-SONAM, an informal mentorship culture existed before the formal establishment of the mentorship program, as indicated within the contextual subtheme, “Existence of mentorship culture". There are numerous benefits of informal mentorship however formal mentoring programs provide opportunities for connection, collaboration and mutual support for both the mentors and mentees [11]. The subtheme ‘Origins and Evolution of Mentoring’ hereby reflects the transition from informal to formal mentoring at AKU-SONAM through the development of a pool of mentors, the selection of mentors who could help in their respective domains followed by the development of both mentors and mentees [12, 13].

The exploration revealed that mentors aimed to nurture skills and expertise in their mentees so that they could become independent and pursue their challenges on their own. They therefore acted in a way that their lives could become a roadmap of exemplary guidance to their mentees. The mentors believed that mentees could learn more by observing them instead of receiving merely a set of instructions. Rehman et al in their study have documented the positivity created by the mentors while they embark on the journey to guide the young ones [14]. The findings “*Role modeling becomes more important in mentoring a faculty since you are preparing someone*, *who in turn will take a leadership role”* corroborates the importance of role models in the literature [15]

The role of social support from peers in the mentorship program was also highlighted by the impact of social interaction on the psychological implications of mentoring [16]

To incentivize their participation, some formal recognition of mentors may be useful.

A well-structured mentorship program, designed to support mentors of diverse age groups, is essential for higher education institutions [17]. ‘Mentoring of Seniors’ concept was emphasized by the foundation of a mentorship exchange program where seniors are being mentored by international scholars and renowned mentors in their respective fields.

The subtheme, ‘Availability of resources’, mentioned a few challenges that are part and parcel of any program. As all successful projects need funding, mentoring also needs monetary resources. Financial support is required not only at the start of the program but also at different stages therefore evaluation of financial needs should be assessed periodically for continual support and sustainability [18] A study conducted in Japan has also emphasized the importance of having ample resources to maintain the work-balance and positive environment among the mentees [19].

The feedback process requires that both faculty members and mentees are being heard and their feedback is taken at regular intervals. One of the challenges faced by the mentors was that the mentoring feedback system was not structured. According to the mentors, guidelines should be clear regarding the assessment for the development of the mentee. Similar to our study, Mubuuke et al reported that feedback from mentors was not in due time, the majority of the mentees did not receive feedback from their mentors and few received only negativity from their mentors [16]

The theme; “Synergies in Professional Development: From Interdepartmental Collaboration to Mentor-Mentee Connections” discusses Mechanisms in the CMO framework

Literature suggests best practices for mentor-mentee matching are consideration of education level, the field of study, and common areas of research [20]. At AKU-SONAM, a pool of available mentors was discussed with the mentees at the time of hiring. The mentors were assigned to the mentees by the committees’ head according to their preference and relevance of the field of the mentor’s specialty. The matched pair was revisited after 4 to 6 weeks to see if they could continue their journey or not and to ensure a positive relationship between the two. Graham in her research has mentioned that the pairing must be reevaluated from time to time. to ensure the satisfaction of the mentoring pair [21]. A match between the mentor’s formal qualifications and the mentees’ skill gaps can help support skill development in the process of mentoring [22]. In the journey of mentorship, the empirical learning and knowledge gained equip mentees with the skills required for excellence in their profession [23]. The ‘Dynamics of Mentor-Mentee Relationship’ can however be strengthened by the quality time dedicated by the mentors [24].

The shared interest between mentors and mentees fosters a mutually beneficial exchange of ideas, emphasizing a collaborative relationship rather than one where seniors dictate to juniors [25].‘Mentors and mentee dynamics’ influence the mentor’s attitudes and behaviors which ultimately foster enthusiasm for mentoring and the effectiveness of mentorship programs [26]. Both the mentor and novice faculty are seated closer for continuous supervision for learning of the mentee in the ‘Work Driven Model’ at AKU-SONAM [27]. Further to the seating arrangement, the study emphasized the need for a bi-directional relationship for the cultivation of reciprocal growth and development of both mentees and mentors [28].

The concept of ‘power mentoring’ involves the participation of different members of the university to uplift the morale and courage of young faculty needing support under the umbrella of mentorship [29]. Reciprocal e-mentoring relationships between professionals from different universities and countries hence can deliver prospects for learning and collaboration [30]. This perspective was outlined in the subtheme **‘**Fostering Interdepartmental Collaboration and Building Connections’ describing the expertise of scholars and seniors as a source of learning enhancement as well as moral support for the mentees.

The outcome theme, ‘*Cultivating Excellence in the Orchard of Mentorship’* signifies that for the success of any project, all efforts should be aligned with the related outcomes. The subtheme, ‘Exchange of Insights in the Orchard of Mentoring’ mentions that “*If we can have an exchange of mentors*, *between SONAM*, *medical college*, *DED and IED which is likely to happen in future we can select a mentor from another entity as per our needs”*. The exchange of ideas and philosophies among the mentors forms the basis for the continuous support offered by the mentors fostering the idea of continual growth both for mentors and mentees [31].

The subtheme, ‘Skills development’ signifies the importance of developing novel competencies at the end of a mentoring program. The mentors believe that the mentoring program at AKU is deficient in monitoring the skills obtained as evidenced by the comment, ‘*We don’t have a formal tool to assess the process of learning or to gauge the learning of mentees on an individual basis*.*’* Designing tools to assess the learning of skills is essential to know the success of the mentoring program. The tools vary according to the nature of mentoring and the fields of mentors and mentees [31, 32]. Research and development was one of the aims that were kept in mind while the program was launched. “*The research office provides mentorship and guidance to all the faculty members*”. A study conducted in the Nursing Science Department in Germany has also emphasized the importance of research and development through mentoring [32]. Formal mentoring programs can foster an environment in which mentees can develop their capacities in the field of innovation and research. The research themes can be designed that cater interdisciplinary approaches [32, 33].

### Limitations

Though the study offers valuable insights into the mentorship program at AKU-SONAM, the reliance on self-reported data with a relatively small sample size potentially limits the transferability of the findings to other institutions. Furthermore, the evaluation of the program’s effectiveness may be subjective, as it relies on qualitative data and perceptions of a few participants. Additionally, the short duration of the formal mentorship program limited the ability to assess its long-term outcomes.

### Conclusion

A strong informal mentorship culture existed prior to the formal program’s establishment, facilitating its implementation at AKU-SONAM. The program fostered a collaborative and supportive relationship with mutual benefits for both mentors and mentees. The program provided social support, contributing to the overall well-being and psychological health of mentees. Mentors served as role models, imparting knowledge and skills to mentees, ultimately enhancing their professional capabilities. However, resource constraints, unstructured feedback mechanisms, and limited mentor availability have been identified as challenges faced by the program.

### Recommendations

To ensure the long-term success of the mentorship program, it is essential to integrate mentoring into the institution’s culture, strategically plan for the recruitment and retention of experienced faculty as mentors, implement a rigorous matching process to optimize mentor-mentee pairings, allocate sufficient resources to support program activities and establish a formal recognition system to acknowledge and reward mentors’ contributions. By taking these steps, the institution can foster a supportive and collaborative environment that empowers both mentors and mentees to achieve their full potential. Moreover, it is crucial to conduct regular evaluations to assess the program’s context, mechanisms, and outcomes. This ongoing monitoring will enable the identification of areas for improvement and the implementation of necessary adjustments to optimize the program’s effectiveness.

Our study has highlighted the context and mechanisms of the CMO framework in the mentoring program at AKU-SONAM, underlining the processes of how it can be implemented along with the fruitful impact of it on the employees and the organization as a whole. However, there remains a need for future research that can encompass the variability in the perception of both mentees and mentors

### Disclaimer

Part of a dissertation for Masters in Health Professions Education

## Abbreviations

CMO: Context, mechanism, and outcome
C: contextual factors
M: mechanisms
O: Outcomes
AKU-SONAM: Aga Khan University School of Nursing & Midwifery
IDI: in-depth interviews
FGDs: focus group discussions
RE: Realistic Evaluation
